# Supplementary material for: Polyketide synthase gene clusters in the ericoid mycorrhizal fungus Oidiodendron maius: genome-wide prediction, expression profiling, and functional characterization of knockout mutants
Source: Front Plant Sci. 2026 Sep 16;17:1924430. doi: 10.3389/fpls.2026.1924430 (PMC13623903; doi:10.3389/fpls.2026.1924430)
Supplement: Supplementary file 1 [file SupplementaryFile1.zip › Supplementary Materials/Supplementary Folder 1.zip!/Oidma1_AssemblyScaffolds_Repeatmasked/knownclusterblast/cluster42/jgi.p_Oidma1_171122_mibig_hits.html]

| MIBiG Protein | Description | MIBiG Cluster | MiBiG Product | % ID | % Coverage | BLAST Score | E-value |
| --- | --- | --- | --- | --- | --- | --- | --- |
| ADM79461.1 | O-methyltransferase | BGC0001266 | Grayanic\_acid\_biosynthetic\_gene\_cluster | 39 | 100.250626566 | 287 | 1.8e-77 |
| ADI24954.1 | GsfB | BGC0000070 | Griseofulvin\_biosynthetic\_gene\_cluster | 33 | 105.263157895 | 217 | 4e-56 |
| BAH23997.1 | O-methyltransferase | BGC0000356 | Fumitremorgin\_biosynthetic\_gene\_cluster | 27 | 83.9598997494 | 136 | 6.8e-32 |
| OAQ83753.1 | sterigmatocystin\_8-O-methyltransferase | BGC0001358 | Leucinostatins\_biosynthetic\_gene\_cluster | 38 | 45.3634085213 | 120 | 5.1e-27 |
| ACS83775.1 | O-methyl\_transferase | BGC0000604 | GE2270\_biosynthetic\_gene\_cluster | 29 | 72.4310776942 | 81 | 3.4e-15 |
